# Supplementary material for: Chromosome-level assembly of Cucumis sativus cv. ‘Tokiwa’ as a reference genome of Japanese cucumber
Source: Breed Sci. 2025 Mar 27;75(2):85–92. doi: 10.1270/jsbbs.24066 (PMC12395194; doi:10.1270/jsbbs.24066)
Supplement: Supplementary file 1 — Supplemental Figure [file 75_085_s1.pdf]

doi: 10.1270/jsbbs.24066

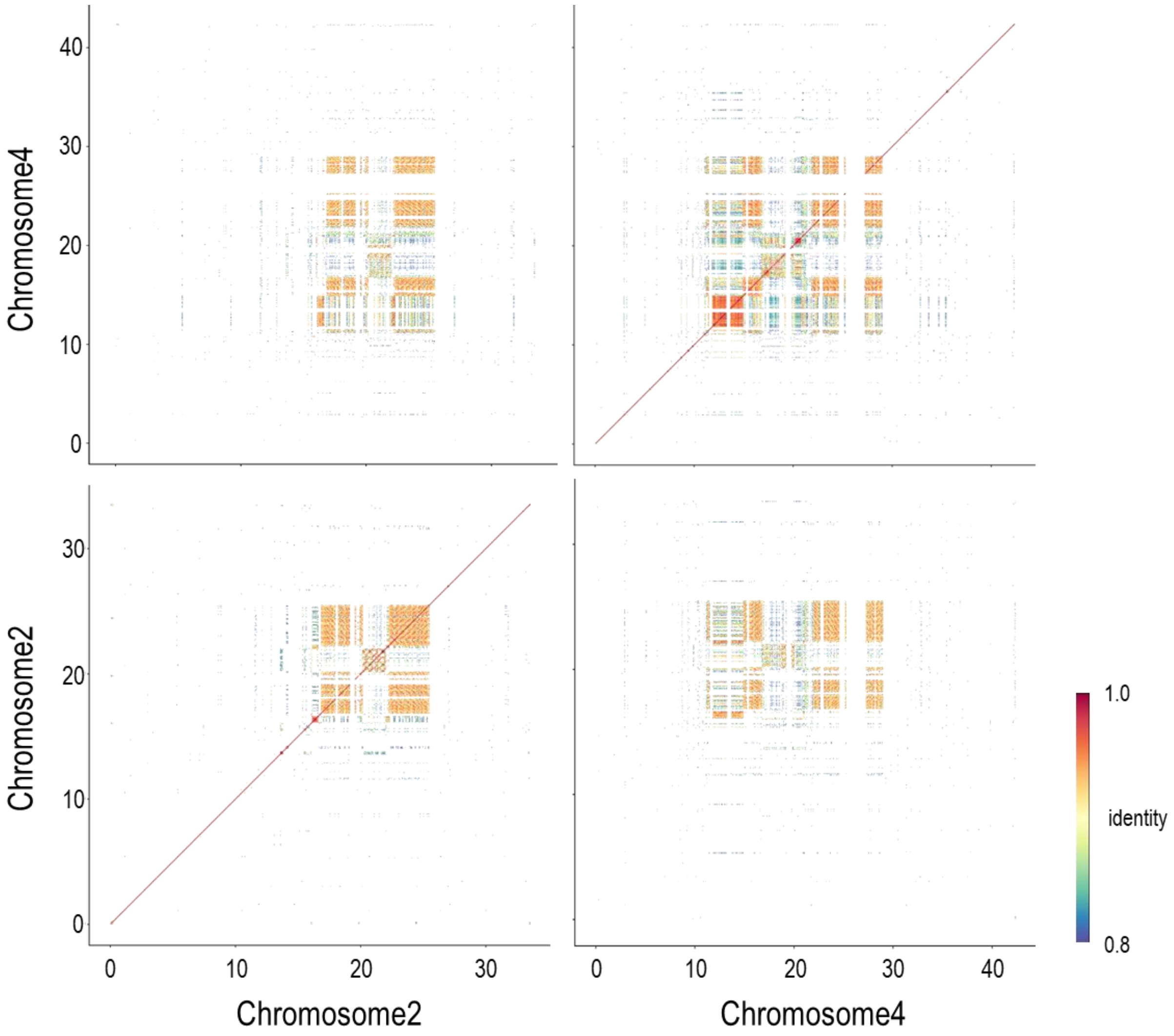

Supplemental Figure 1. Comparative dot plots of chromosome2 and chromosome4, with 80% identity threshold. The color scale indicates degree of identity between the sequences compared. Scales in X- and Y- axes are indicate in million base pairs.
